# Case Report: Obstructive sleep apnea syndrome-associated blood pressure fluctuations combined with simultaneous central retinal vein and artery occlusion

**DOI:** 10.3389/fmed.2025.1614489

**Published:** 2025-08-11

**Authors:** Zhenzhen Gu, Jinhua Luo, Xi Chen, Kaiming Chen, Pin Ju, Mei Sun, Xiaofeng Hao, Like Xie

**Affiliations:** ^1^China Academy of Chinese Medical Sciences, Beijing, China; ^2^Eye Hospital, China Academy of Chinese Medical Sciences, Beijing, China

**Keywords:** central retinal vein occlusion, central retinal artery occlusion, obstructive sleep apnea syndrome, retinal vascular occlusion, hypoxemia hemodynamic disturbances

## Abstract

**Background:**

Central retinal vein occlusion (CRVO) and central retinal artery occlusion (CRAO) are serious eye blood vessel problems usually linked to heart health issues. In this case study, a patient with a diagnosis of obstructive sleep apnea syndrome (OSAS) but no traditional thrombotic or atherosclerotic risk factors experienced a rare co-occurrence of CRVO and CRAO.

**Case presentation:**

A formerly healthy 42-year-old man came with dark shadows in his right eye and acute-onset blurred vision. A thorough ophthalmic exam verified simultaneous CRVO and CRAO. Later, polysomnography showed moderate hypoxemia and severe OSAS. The patient received integrated traditional Chinese medicine, perfusion enhancement, and intraocular pressure lowering as part of multimodal therapy. With notable retinal edema resolution, post-treatment visual acuity returned to 20/20.

**Conclusion:**

Especially in patients without conventional cardiovascular risks, this case implies that OSAS might be a separate risk factor for combined CRVO and CRAO. Intermittent hypoxia, sympathetic overactivity, and hypercoagulability are probably among the underlying mechanisms. Optimizing management plans in idiopathic retinal vascular occlusions seems to depend on early OSAS screening. Moreover, this case shows the possible therapeutic benefit of combining pharmacological and conventional treatments for complicated ocular vascular disorders.

## 1 Introduction

The simultaneous presence of central retinal artery occlusion (CRAO) and central retinal vein occlusion (CRVO) constitutes a rare vascular event, usually showing with painless vision loss. Generally, a fundoscopic exam shows retinal hemorrhages, ischemic whitening, optic disk hyperemia and edema, venous dilatation and tortuosity, cotton wool spots, and Roth spots (1). In combined vascular occlusions, the prognosis is usually poor and often complicated by rubeosis iridis and neovascular glaucoma (2). Clinical research has shown a significant association between retinal vascular diseases and both obstructive sleep apnea syndrome (OSAS) and uncontrolled hypertension (3). OSAS is a common but often overlooked sleep-related breathing disorder. Several pathological mechanisms contribute to airway narrowing or collapse during sleep, leading to upper airway obstruction, increased breathing effort, and poor ventilation, which in turn promote hypertension and fragmented sleep (4). The clinical symptoms of OSAS include snoring, witnessed apneas, awakenings with a sensation of breathlessness, dry mouth, excessive daytime sleepiness, and cognitive impairment (5). This case study describes a rare occurrence of combined CRVO and CRAO likely triggered by blood pressure fluctuations associated with OSAS.

## 2 Case report

A 42-year-old man was admitted to our hospital in March 2024, presenting with abrupt, painless vision loss and a dark shadow obstructing his right eye for 1 week. He reported no prior ocular or medical history and was not taking any medications.

**Figure 1:**
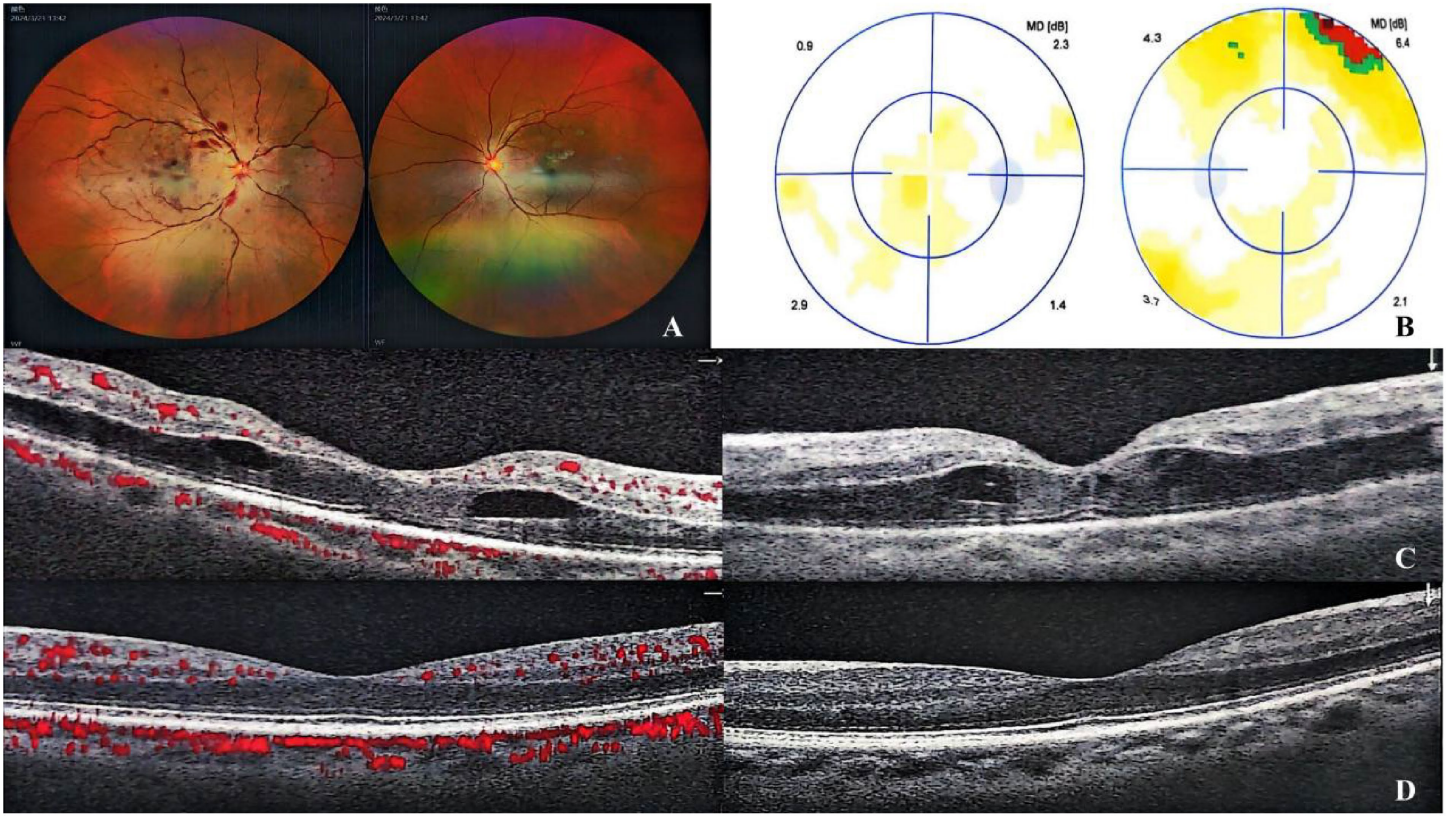
Fundus photography revealed tortuous and dilated retinal veins with attenuated retinal arteries in the right eye. Scattered dots and blot hemorrhages were observed along the vascular arcades, predominantly in the posterior pole, with macular involvement. Flame-shaped hemorrhages were noted in the superior and inferior temporal branches **(A)**. Visual field testing showed no significant abnormalities **(B)**. OCTA of the right eye demonstrated macular edema **(C)**. The left eye exhibited no significant pathological changes **(D)**.

**Figure 2:**
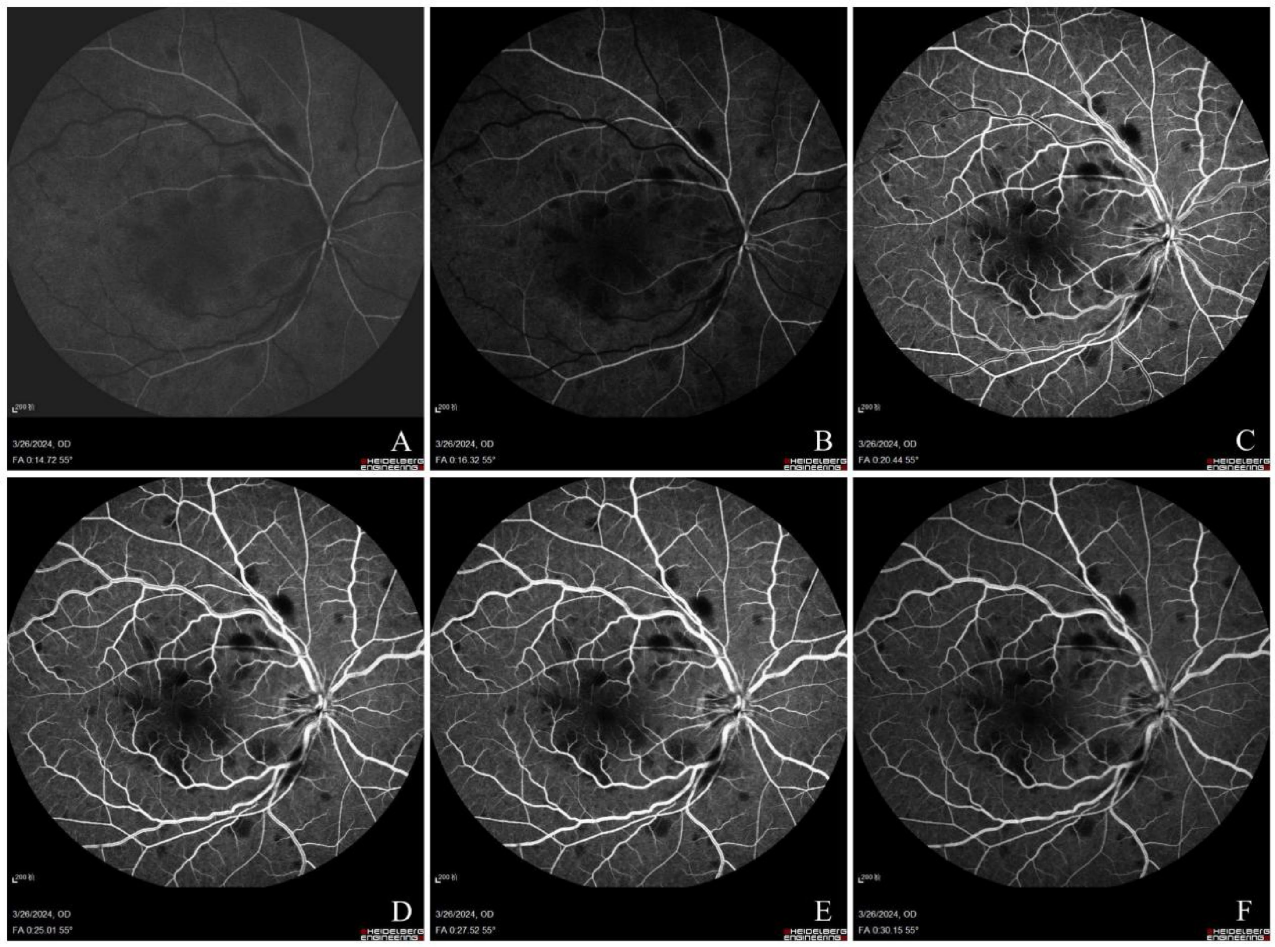
Arterial phase: delayed filling of perimacular arterioles with attenuated retinal arteries **(A)**. Early venous phase: delayed filling of the central retinal vein with superficial optic disk capillary dilation **(B)**. Venous phase: tortuous and dilated veins with mild vessel wall hyperfluorescence. Scattered patchy hemorrhages (blocked fluorescence) predominantly in the posterior pole, showing significant non-perfusion (NP) areas. Dot hyperfluorescence (transmitted fluorescence) observed in the temporal periphery **(C–E)**. Late venous phase: mild macular leakage **(F)**.

At presentation, his best-corrected visual acuity (BCVA) was 20/100 in the right eye and 20/25 in the left eye. The intraocular pressure was 18 mmHg in his right eye and 15.5 mmHg in his left eye. Fundus photography revealed tortuous and dilated retinal veins in the right eye, thin retinal arteries, and patchy hemorrhages along the course of the vessels, which were more prominent in the posterior pole and involved the macular area (Figure 1A). The right eye was normal. Visual field examination showed no abnormalities (Figure 1B). Optical coherence tomography angiography (OCTA) demonstrated macular edema in the right eye (Figure 1C), while the left eye showed no abnormalities (Figure 1D). Fluorescein angiography showed delayed filling of the central retinal vein and perimacular arterioles. Early-phase imaging revealed dilation of superficial capillary networks at the optic disk, attenuated retinal arteries, and tortuous venous dilation, accompanied by mild vascular wall hyperfluorescence. Scattered patchy hemorrhages causing fluorescence blockage were predominantly observed in the posterior pole, along with a prominent non-perfusion area. Dot-like transmitted fluorescence, suggestive of retinal pigment epithelial alterations, was detected in the temporal periphery. Late-phase imaging revealed mild fluorescein leakage in the macular region. The arterial phase duration was 14.72 s, the arteriovenous phase was 1.6 s, and the venous filling time was 19.53 s—findings consistent with CRVO and incomplete CRAO in the right eye (Figure 2).

His electrocardiogram, carotid ultrasound, and echocardiographic examinations showed no abnormalities. Laboratory tests, including hemogram, hemorrheology, biochemistry, coagulation profile, random blood sugar, and urinalysis, also showed no abnormalities. The patient was diagnosed with CRVO combined with central retinal artery occlusion CRAO in the right eye, based on his symptoms, clinical indicators, and thorough ophthalmological examination.

Further medical history was obtained to rule out cardiovascular risk factors. The patient was a non-smoker with no history of hypertension, diabetes, hyperlipidemia, hyperhomocysteinemia, hyperuricemia, cerebrovascular accident, ischemic heart disease, systemic vasculitis, recent dental surgery, or facial cosmetic surgery. He was not taking any medications and had no family history of thromboembolism. Upon admission, his blood pressure was 137/82 mmHg. Ambulatory blood pressure monitoring indicated an average daytime blood pressure of 145/96 mmHg and an average overnight blood pressure of 125/87 mmHg.

After a comprehensive assessment and detailed review of the patient’s medical history, it was noted that he experienced snoring during sleep, with symptoms worsening over the past 6 months. His body mass index (BMI) was 31.14 kg/m^2^. Endoscopic examination revealed a narrowed posterior pharyngeal space. Subsequent sleep monitoring confirmed severe obstructive sleep apnea-hypopnea syndrome, accompanied by mild hypoxemia. The apnea–hypopnea index was 46.5, with a minimum oxygen saturation of 82%. Given the activation of the right sympathetic nervous system and the patient’s currently stable blood pressure, no antihypertensive medication was prescribed at this time.

The patient was treated with carteolol hydrochloride eye drops and underwent right eye anterior chamber paracentesis to decrease intraocular pressure and enhance retinal artery perfusion. He received oxygen for 6 h daily to dilate his blood vessels. For macular edema, a dual therapy regimen was administered, consisting of a periocular injection of dexamethasone sodium phosphate and an intravitreal injection of 0.05 mL ranibizumab, targeting both the surrounding and intraocular regions. The comprehensive treatment plan of traditional Chinese medicine (TCM) consisted of intravenous Xuesaitong injection (XST, 300 mg daily) and Puerarin injection (200 mg daily) to promote vasodilation and improve microcirculation. The patient also received oral herbal medicine treatment to enhance blood circulation in the eyes. The prescribed herbal formula consisted of stir-fried Semen Persicae (Taoren), Chuanxiong Rhizome (Chuanxiong), Carthami Flos (Honghua), Rehmanniae Radix (Shengdi), Gallus Gigerii Endothelium Corneum (Jineijin), Pinelliae Rhizoma Praeparatum (Fabanxia), Citri Reticulatae Pericarpium (Chenpi), Angelicae Sinensis Radix (Danggui), Pheretima (Dilong), Poria (Fuling), Notoginseng Powder (Sanqifen), and Saposhnikoviae Radix (Fangfeng). These integrated treatment modalities were utilized to enhance blood circulation and alleviate blood stasis.

**Figure 3:**
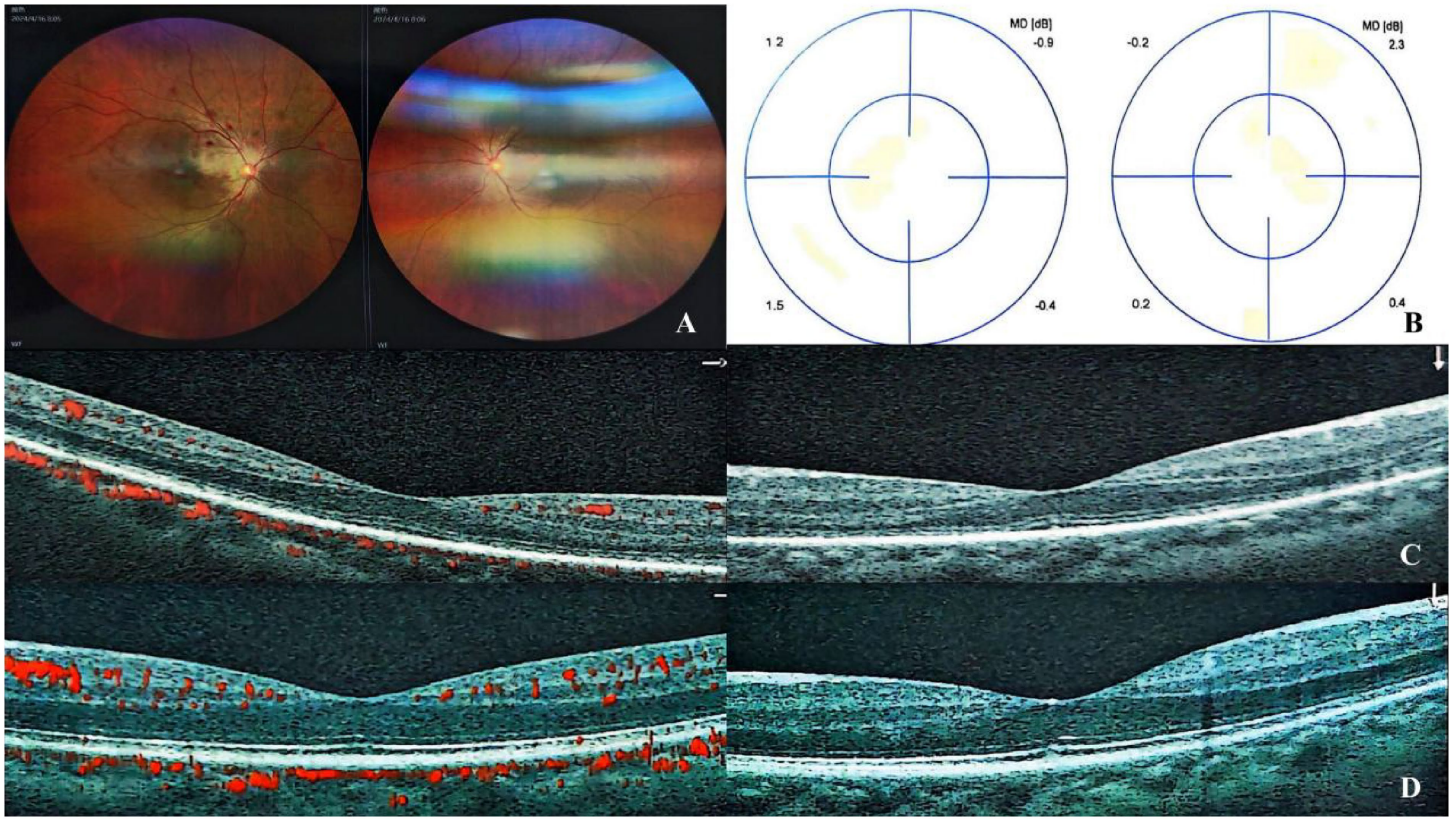
Fundus examination revealed partial absorption of retinal hemorrhages with a normalized vascular architecture and improved venous tortuosity compared to previous findings **(A)**. Visual field testing showed no significant defects **(B)**. OCTA demonstrated decreased vascular density in the right eye, accompanied by marked resolution of retinal edema **(C)**, while the left eye remained stable **(D)**.

**Figure 4:**
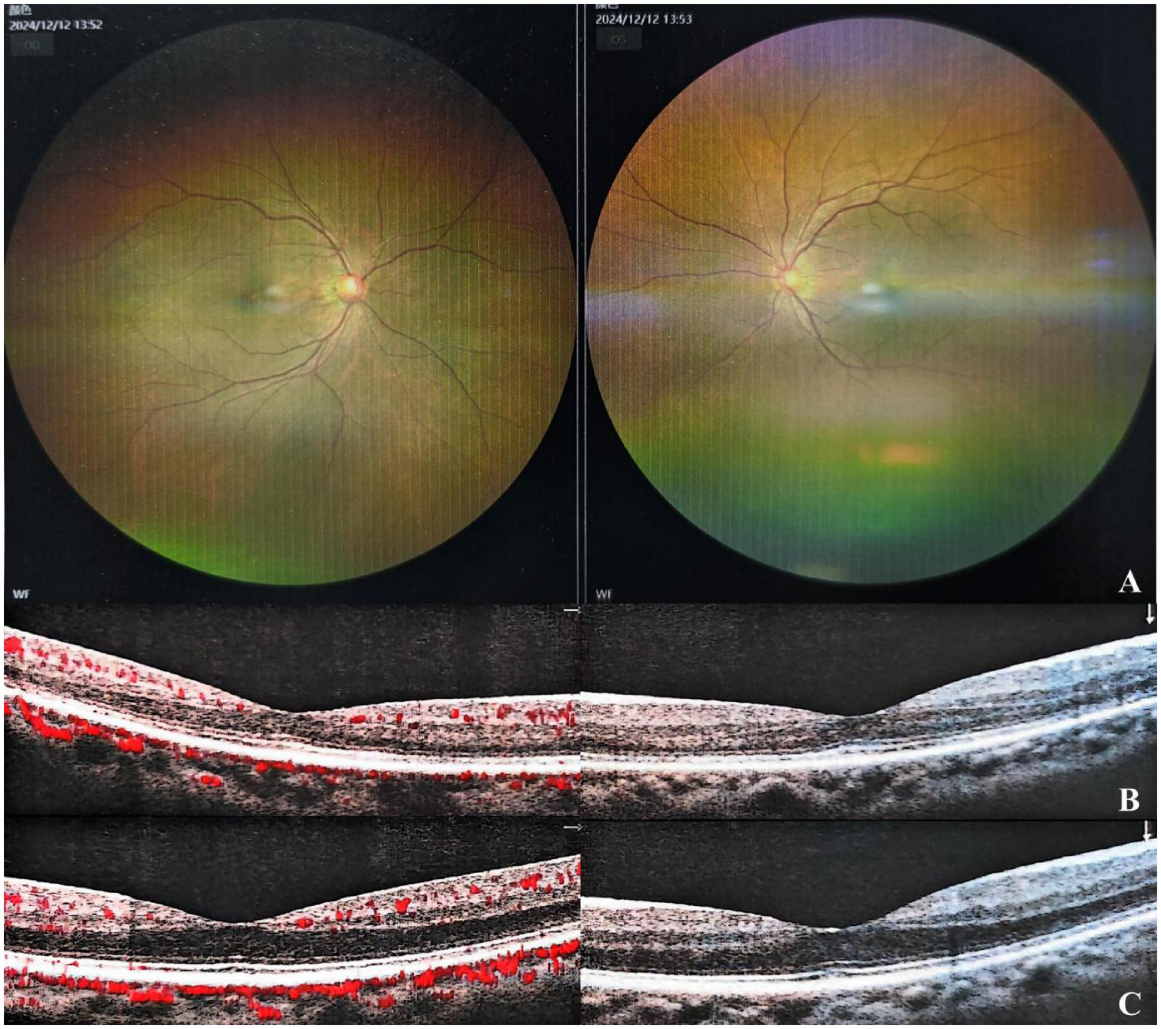
Fundus examination revealed no abnormalities in both eyes **(A)**. OCTA showed normal findings in the right **(B)** and left eyes **(C)**.

During the initial assessment, 2 weeks post-treatment, his uncorrected visual acuity in the right eye improved to 20/40, with his best-corrected visual acuity achieving 20/20. The intraocular pressure in the patient’s right eye is 13.1 mmHg, while the pressure in the left eye is 15.7 mmHg. His fundus photography showed that the hemorrhage in his right eye had absorbed more than before, the blood vessels were normal, and the tortuosity of the veins had improved (Figure 3A). OCTA demonstrated reduced vascular density in the right eye with significant resolution of retinal edema (Figure 3C), while visual field testing revealed no detectable defects in the right eye (Figures 3B,D). The patient reported subjective improvement in the right eye, describing that the previously noted dark shadow in the visual field had become smaller in size. At 8-month follow-up, his vision remained the same as before, intraocular pressure was normal, and there were no abnormalities in the fundus and OCT (Figure 4).

## 3 Discussion

In this unique case, the co-occurrence of OSAS, CRVO, and CRAO represents a rare event that has not been extensively studied. Most patients with OSAS are associated with multiple cardiovascular complications, including hypertension, atrial fibrillation and other arrhythmias, heart failure, coronary artery disease, stroke, pulmonary arterial hypertension, metabolic syndrome, and diabetes (6).

The patient’s relatively preserved visual acuity (20/100) despite CRAO may be attributed to the incomplete nature of arterial occlusion and the coexisting CRVO, which potentially created partial retrograde perfusion that maintained some retinal function. In this middle-aged male with a simple medical history, initial clinical and laboratory examinations ruled out traditional cardiovascular risk factors, suggesting that OSAS may be an independent risk factor for retinal vascular events. This is consistent with existing evidence that OSAS and uncontrolled hypertension significantly increase the risk of retinal vein occlusion (RVO) (7, 8). Severe OSAS in patients may lead to retinal microcirculatory disorders through multiple mechanisms, including intermittent hypoxia, hypercapnia-induced sympathetic nervous system overactivation, systemic oxidative stress, and hypercoagulability. These factors collectively impair retinal blood flow perfusion and exacerbate vascular endothelial damage, consistent with the pathophysiological mechanisms proposed in previous studies on OSAS-RVO (9). Hee Jung Kwon et al. reported that OSAS may be a unique risk factor in the pathogenesis of branch retinal vein occlusion (BRVO) in patients without comorbidities such as hypertension and diabetes (10). As in our patient’s case, the patient lacked traditional thrombotic or atherosclerotic risk factors, further suggesting that retinal lesions may be the early clinical manifestation of undetected OSAS (11). Therefore, appropriate sleep evaluation is crucial for patients with newly diagnosed retinal vascular disease.

In terms of treatment, comprehensive interventions targeting vascular and inflammatory pathways demonstrate the application of multimodal combination therapy in reducing macular edema and improving retinal perfusion. The use of medications and anterior chamber paracentesis to lower intraocular pressure can dilate retinal arteries and reduce retinal arterial perfusion pressure. This invasive intervention has shown particular efficacy in treating retinal artery occlusion lasting more than 12 h, improving the BCVA of patients and demonstrating good clinical efficacy and safety, as supported by our clinical observations (12). Anti-VEGF therapy eliminates edema, improves vision, and reduces non-perfused retinal areas, thereby preventing neovascular complications (13). Arrigo et al. reported a case of a 51-year-old man with concurrent retinal venous and arterial occlusion who responded well to dexamethasone injection therapy, with complete and sustainable recovery of ocular function (14). As in our patient, dexamethasone injection therapy can reduce macular edema, thereby aiding in improved arterial perfusion.

The patient received comprehensive TCM treatment during hospitalization. XST, which is widely used in the clinical treatment of retinal vein obstruction, can improve microcirculation and hemorheology (15). Studies have shown that XST reduces whole blood high-shear viscosity, whole blood low-shear viscosity, plasma viscosity, and plasma fibrinogen levels while maintaining good patient safety (16, 17). Similarly, Puerarin injection, commonly used in treating retinal artery and vein occlusion (18, 19), is a vasodilator that can expand coronary and cerebral arteries, reduce myocardial oxygen consumption, improve microcirculation, and inhibit platelet aggregation (20). Multiple animal studies have demonstrated that Puerarin injection exerts its therapeutic effects by regulating the VEGF signaling pathway (21). The patient also received oral Chinese herbal medicine treatment. This TCM formula has been confirmed to inhibit the expression of HIF-1α and VEGF mRNA in retinal tissues (22), which provides a basis for understanding its mechanism of action. Unlike the single-agent anti-VEGF therapy mainly targeting angiogenesis, the combined treatment with TCM has a multi-target synergistic effect. An animal experiment also demonstrated that TCM for promoting blood circulation and improving eyesight can alleviate retinal edema in BRVO models and rescue retinal structure and function by promoting recanalization of occlusive veins, improving microcirculation, and regulating the expression of VEGF-α (23). A meta-analysis indicates that anti-VEGF therapy combined with TCM provides superior clinical outcomes compared to anti-VEGF therapy alone, demonstrating benefits such as reducing the number of injections and increasing the total effective rate (24). This multi-target strategy may explain why, in this case, only one anti-VEGF injection was needed to control macular edema, while patients receiving standard Western medicine treatment typically require four injections to achieve the same level of edema control effect (25). However, while these findings are promising, the long-term efficacy of TCM combined with conventional treatment requires further investigation through larger clinical trials and longer follow-up periods.

It is worth noting that during hospitalization at an ophthalmic specialty center, patients received only health education—such as guidance on weight control, smoking cessation, and sleep position adjustment—and did not undergo standardized treatment for OSAS, such as continuous positive airway pressure (CPAP) therapy. This was primarily due to institutional limitations in CPAP availability and issues with patient compliance during follow-up. As a result, an opportunity to improve vascular regulatory function at the etiological level may have been missed (26). This case highlights the need for stronger collaboration between ophthalmologists and sleep specialists to ensure comprehensive management of OSAS in patients with retinal vascular events. Additionally, it is crucial to note that recent blood pressure fluctuations in patients were associated with severe sleep apnea syndrome. Elevated daytime blood pressure further suggests that OSAS-related hypertension may not have been adequately identified, emphasizing the need to improve patient education on the systemic risks of untreated OSAS and to establish long-term monitoring protocols for such high-risk patients. These findings underscore the importance of conducting systematic evaluations in patients with unexplained retinal vascular occlusion and implementing multidisciplinary approaches for optimal patient outcomes.

## 4 Conclusion

This case underscores the uncommon simultaneous occurrence of CRVO and CRAO in a patient lacking conventional thrombotic or atherosclerotic risk factors yet presenting with underlying OSAS. The results indicate that OSAS may act as an independent risk factor for combined retinal vascular occlusions, especially in younger individuals without traditional cardiovascular comorbidities. The effective visual recovery in this case highlights the significance of early OSAS screening in patients with idiopathic retinal vascular occlusions, and tailored care of sleep-disordered breathing may reduce further vascular problems. Furthermore, multimodal combination therapy—including intraocular pressure reduction, enhanced perfusion, and TCM—has shown promising effectiveness in ameliorating outcomes in intricate ocular vascular diseases. This article underscores the necessity for an exhaustive diagnostic evaluation in unusual instances of retinal vascular occlusions, promoting a multidisciplinary strategy that includes ophthalmologists, sleep experts, and internists to enhance patient care and avert irreversible visual impairment. Future research should investigate the molecular connections between obstructive sleep apnea and retinal vascular incidents to enhance therapeutic approaches.

## Data Availability

The raw data supporting the conclusions of this article will be made available by the authors, without undue reservation.
